# Supplementary material for: Electrospray Deposition of Cellulose Nanofibers on Paper: Overcoming the Limitations of Conventional Coating
Source: Nanomaterials (Basel). 2021 Dec 29;12(1):79. doi: 10.3390/nano12010079 (PMC8746688; doi:10.3390/nano12010079)
Supplement: Supplementary file 1 [file nanomaterials-12-00079-s001.zip › nanomaterials-1482587-supplementary.pdf]

nanomaterials

Supplementary Materials

Electrospray Deposition of Cellulose Nanofibers on Paper: Overcoming the Limitations of Conventional Coating

Quim Tarrés, Roberto Aguado *, M. Àngels Pèlach, Pere Mutjé and Marc Delgado-Aguilar

LEPAMAP-PRODIS Research Group, University of Girona, M Aurèlia Capmany, n°61, 17003 Girona, Spain; joaquimagusti.tarres@udg.edu (Q.T.); angels.pelach@udg.edu (M.À.P.); pere.mutje@udg.edu (P.M.); m.delgado@udg.edu (M.D.-A.)
* Correspondence: roberto.aguado@udg.edu

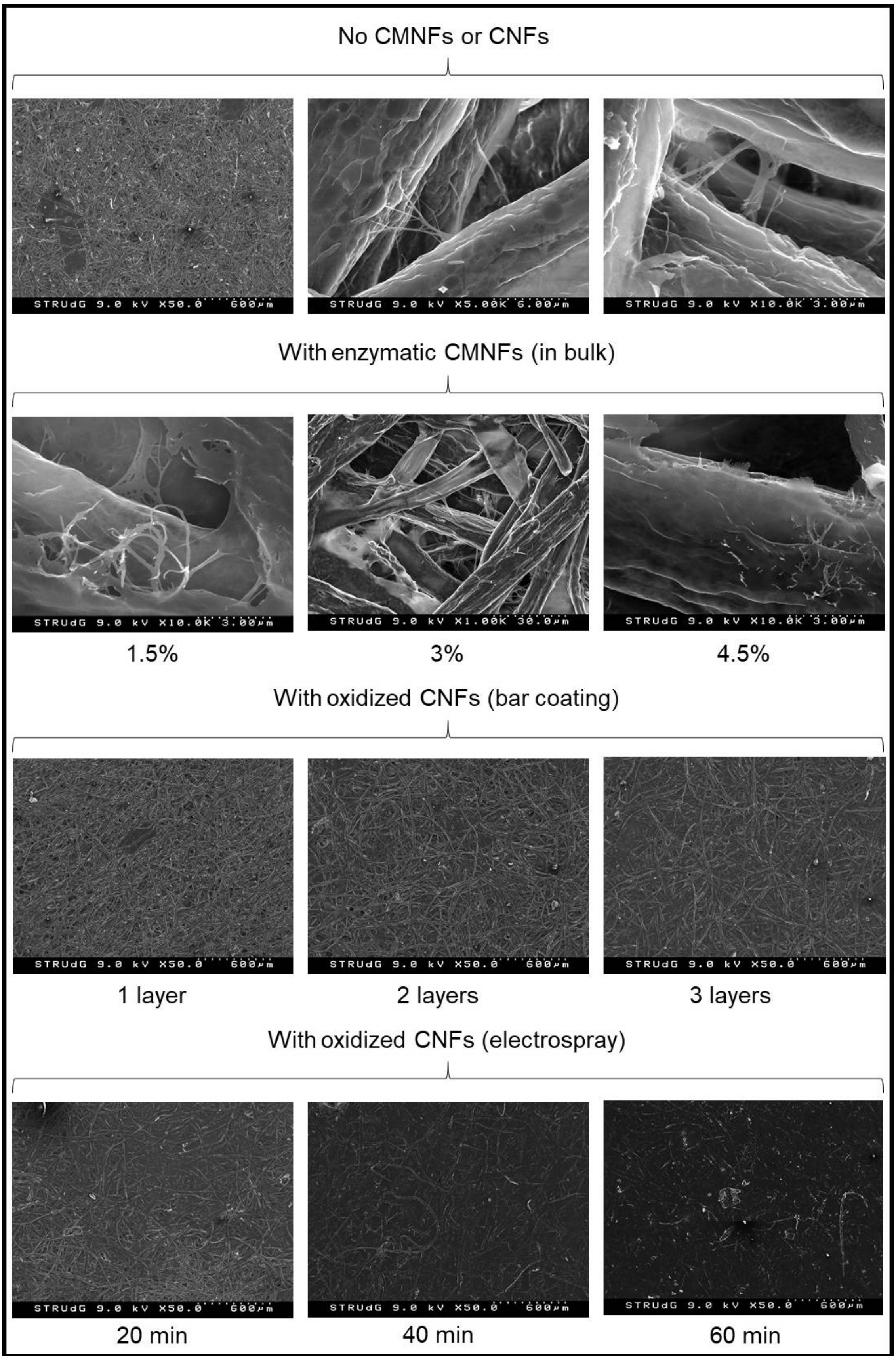

Figure S1. Additional micrographs.
